# Intrinsic and extrinsic apoptosis responses in leukaemia cells following daunorubicin treatment

**DOI:** 10.1186/s12885-021-08167-y

**Published:** 2021-04-21

**Authors:** Hussain Mubarak Al-Aamri, Helen R. Irving, Christopher Bradley, Terri Meehan-Andrews

**Affiliations:** 1 Department of Pharmacy and Biomedical Science, La Trobe Institute for Molecular Science (LIMS), La Trobe University, P.O. Box 199, Bendigo, VIC 3552 Australia; 2 Oman College of Health Sciences, PO Box 293, 620 Ruwi, Sultanate of Oman

**Keywords:** Daunorubicin, Apoptosis, Leukaemia, Mitochondrial membrane potential, Caspases

## Abstract

**Background:**

Daunorubicin is used clinically in the treatment of myeloma, acute lymphatic and myelocytic leukaemia. The toxic lesions caused by daunorubicin induce various modes of cell death, including apoptosis. Apoptosis is highly regulated programmed cell death that can be initiated mainly via two pathways, through death receptors (extrinsic) or involvement of the mitochondria (intrinsic). Induction of apoptosis via these pathways has been alluded following treatment with daunorubicin, but never compared in acute lymphoblastic leukaemia over a time course.

**Methods:**

This study investigated the mechanisms of daunorubicin induced apoptosis in the treatment of CCRF-CEM, MOLT-4 (acute T-lymphoblastic leukaemia) and SUP-B15 (acute B-lymphoblastic leukaemia) cells. Cells were treated with daunorubicin for 4 h, and then placed in recovery medium (without daunorubicin) for 4 h, 12 h and 24 h. Apoptotic response was analysing using annexin-V expression, caspase activity, mitochondrial membrane potential change and an array to detect 43 apoptotic proteins.

**Results:**

Daunorubicin induced apoptosis in all leukemic cell lines, but with different levels and duration of response. Both apoptosis levels and caspase activity increased after four hours recovery then declined in CCRF-CEM and MOLT-4 cells. However, SUP-B15 cells displayed initially comparable levels but remained elevated over the 24 h assessment period. Changes in mitochondrial membrane potential occurred in both MOLT-4 and CCRF-CEM cells but not in SUP-B15 cells. Expression of apoptotic proteins, including Bcl-2, Bax, caspase 3 and FADD, indicated that daunorubicin potentially induced both extrinsic and intrinsic apoptosis in both CCRF-CEM and MOLT-4 cells, but only extrinsic apoptosis in SUP-B15 cells.

**Conclusions:**

This study describes variations in sensitivities and timing of apoptotic responses in different leukaemia cell lines. These differences could be attributed to the lack of functional p53 in coordinating the cells response following cytotoxic treatment with daunorubicin, which appears to delay apoptosis and utilises alternative signalling mechanisms that need to be further explored.

**Supplementary Information:**

The online version contains supplementary material available at 10.1186/s12885-021-08167-y.

## Background

Apoptosis is a fundamental mechanism of cell death that is engaged by a range of internal and external factors. One action of chemotherapeutic drugs is to eliminate and remove damaged cells via activation of apoptosis. Understanding how the cell death program is engaged following treatment, and why it fails to be initiated in certain cancer types, is crucial in overcoming clinical problems of drug resistance. Daunorubicin is an anthracycline antibiotic which is widely used clinically in myeloma, acute lymphoblastic leukaemia (ALL) and myeloid leukemias [1] and has a broad range of therapeutic mechanisms with several toxic outcomes, and thus can potentially induce various types of cell death involving different regulatory pathways. Daunorubicin induced apoptotic and necrotic death of acute leukaemia cells through changes in mitochondrial membrane potential (Δψm) and reactive oxygen species (ROS) generation, which promoted mitochondria membrane permeabilization and subsequent induction of apoptosis [2]. The effect of daunorubicin on leukemic cells is caused by both blocking the cell cycle at G2 phase and induction of cell death [3]. Daunorubicin also induces morphological changes such as cell shrinkage and nuclear condensation, which are typical characteristics of apoptosis [4]. In addition, the clinically effective doses of daunorubicin vary depending on the type of leukaemia [5] but the reason for this difference is not clear. It could be due to varying cellular levels of apoptotic proteins, or how daunorubicin impacts on the expression of other proteins, which could also lead to chemo-resistance over time. To unravel the exact pathway of apoptosis in response to daunorubicin, it is necessary to look more closely at the intracellular mechanisms being activated.

Apoptosis is often referred to as a process of cell suicide since the cells own proteins participate in its demise. Chemotherapy drugs induce damage, and it is the balance between pro- and anti-apoptotic signals that ultimately decides the cells fate. The mechanisms initiating apoptosis include both intrinsic and extrinsic signals, are complex and there is considerable crosstalk between them. The intrinsic pathway of apoptosis, also known as the mitochondrial pathway of apoptosis, is initiated following DNA damage, activation of p53, triggering a cascade of events leading to mitochondrial outer membrane permeabilization (MOMP). This leads to Δψm dissipation, arrest of mitochondrial ATP synthesis and Δψm dependent transport activities [6]. The intrinsic pathway is tightly regulated by members of the Bcl-2 family of proteins, which includes both pro-apoptotic and anti-apoptotic proteins. Heterodimeric interactions between the Bcl-2 proteins, via Bcl-2 homology domains (BH1, − 2, − 3 and − 4), ultimately determine the cells fate. The anti-apoptotic proteins within the Bcl-2 family include: Bcl-2, Bcl-xl, Mcl-1 and Bcl-w and all have 4 BH domains. Pro-apoptotic members are divided into two subgroups according to the number of BH domains. The Bcl-2 multidomain subfamily includes Bax and Bak and the BH3-only subfamily includes Bid, Bad, Bim, Puma and Noxa [7, 8]. Following activation of the death signal BH3 domain-only proteins can neutralise or derepress anti-apoptotic Bcl-2 proteins allowing pro-apoptotic proteins such as Bax and Bak like proteins to initiate changes in mitochondrial membrane potential [9]. There is a release of proteins normally contained within the mitochondrial inter-membrane space into the cytosol. One of these proteins is cytochrome c, which combines with pro-caspase 9 and APAF-1 to form the apoptosome [10–12]. The apoptosome aids in the activation of caspase 9, which in turn activates the effector caspase 3. Over-expression of the anti-apoptotic Bcl-2 proteins is a characteristic of chronic lymphocytic leukaemia [13, 14].

The extrinsic pathway of apoptosis, the death receptor pathway, involves a subset of TNF receptors (TNFR) including DR3 (Death Receptor 3), Fas, TNF-R1, TRAIL-R1 (Tumor necrosis factor-Related Apoptosis-Inducing Ligand - Receptor 1), and TRAIL-R2. These death receptors, upon ligand engagement, lead to activation of the intracellular signalling intermediaries that ultimately triggers activation of the initiator caspase 8 [15]. Daunorubicin induces the Fas/FasL pathway through upregulation of the Fas receptor and increase in the levels of FasL [16]. When Fas receptor is trimerized by its ligand, FasL, several intracellular adapter proteins are recruited to the clustered receptors. These molecules, known as FADD (Fas Associated Death Domain), bind to intracellular Fas death domains and recruit caspase 8 or caspase 10. This forms a supramolecular platform referred to as the death-inducing signalling complex (DISC), and responsible for activation of caspase 8 (or 10) [17–19]. Caspase 8 activates caspase 3 by proteolytic cleavage, or alternatively stimulates MOMP by cleavage of Bid, leading to generation of a mitochondrial-permeabilizing fragment known as truncated Bid [20, 21].

Both the intrinsic and extrinsic pathways converge and activate caspases, which are cysteine proteases that are central regulators of apoptosis. Caspase 3 is one of the most frequently activated proteases involved in apoptosis [22]. Caspase-3 plays a main role in mediating nuclear apoptosis in different cell types. Caspase-3 is required for some typical nuclear and other morphological changes associated with the accomplishment of apoptosis such as cell shrinkage, blebbing, chromatin condensation and DNA fragmentation [23, 24].

This study aims to assess the mechanisms of daunorubicin induced apoptosis in three different leukaemia cells lines: MOLT-4 and CCRF-CEM (acute T-lymphoblastic leukaemia) and SUP-B15 (acute B-lymphoblastic leukaemia). Daunorubicin induced cytotoxicity is predominantly caused by apoptosis following failure of DNA repair or by the direct effect of ROS on mitochondrial function [2]. Apoptosis following cell cycle arrest and failure of DNA repair is mediated by p53-Bax induced release of cytochrome c and APAF1 from mitochondria to form an apoptosome with subsequent activation of caspases. The production of ROS on the other hand causes mitochondrial membrane dysfunction, change in membrane permeability and release of mitochondrial proteins that also lead to apoptosis. The relative timing of these two apoptotic events will be assessed by analysing annexin-V expression, caspase activity and Δψm. The levels of p53, Bax, and other apoptotic proteins following treatment with daunorubicin will be determined using apoptotic arrays.

## Methods

### Cell culture and treatments

CCRF-CEM (T-lymphoblastic leukaemia), MOLT-4 (T-lymphoblastic leukaemia) and SUP-B15 (B-lymphoblastic leukaemia) cell lines were obtained from American Type Cell Culture Collection (ATCC). Cells were incubated at 37 °C in atmosphere of 5% CO_2_. Cells were treated with 10 μM of daunorubicin (Sigma-Aldrich) for 4 h, and then placed in recovery medium (without daunorubicin) for 4 h, 12 h and 24 h before assays [25].

### Annexin-V assay

To distinguish between apoptosis and necrosis Annexin-V / Propidium Iodide (PI) stain was utilised (eBioscience). Cells were seeded at 1 × 10^6^ cells per well in a six well plates. Following treatment, medium was collected and centrifuged. Cells were washed in phosphate buffered saline (PBS) and resuspended in binding buffer. Annexin V-FITC (90 μg/mL) and propidium iodide (1 μg/mL) were added to the cell suspension and kept on ice, in the dark for 15 min at room temperature (RT). Prior to analysis an additional 400 μL of binding buffer was added to each sample, and then assessed by flow cytometry (Accuri® C6, Flow cytometry, Ann Arbor, MI, USA).

### Apostat assay

FITC-conjugated pancaspase inhibitors (Apostat; R&D System, Minnepolis, MN, USA) was used to assess activation of caspases. The cells were plated onto twelve well plates at a seeding density of 1 × 10^6^ cells per well. Cells were stained during the last 30 mins of the recovery period with ApoStat (10 μL per 1 mL culture volume). After the staining period, the cells were centrifuged and washed once in PBS and resuspended in 500 μL of PBS for flow cytometric analysis (Accuri® C6, Flow cytometry, Ann Arbor, MI, USA).

### Mitochondria membrane potential (DiOC6) assay

To quantify the changes in mitochondria membrane potential (Δψm) in living cells the assay was carried out based on the protocol outlined by Chang et al. (2013) [26]. Cells were seeded at 1 × 10^6^ cells per well in six well plates. Following treatment, medium was collected, centrifuged and resuspended in 1 mL of DiOC6 stain (0.1 μM, Enzo) and incubated for 30 min at 37 °C. After incubation, tubes were centrifuged at 130 g for 5 min and the supernatant was removed, cells were washed three times in PBS. Finally, 500 μL of PBS was added and tubes and analysed by flow cytometry, Ex/Em 482/501 nm (Accuri® C6, Flow cytometry, Ann Arbor, MI, USA). A positive control, cells suspended in 4% paraformaldehyde, was included to deplete mitochondrial membrane potential, to confirm experimental conditions were impacting on Δψm.

### Apoptosis antibody Array

An apoptosis array was conducted using a human apoptosis array kit (Abcam, UK) to detect changes in 43 apoptotic related proteins. Following treatment, cells were removed from plates, washed with PBS and solubilized in 2x lysis buffer mixed with 1x protease inhibitor cocktail at 1 × 10^7^ cells/mL for 30 min at 2–8 °C. Lysates were centrifuged at 14,000 x g for 5 min and supernatant samples collected into pre-chilled Eppendorf tubes and kept on ice. The protein concentration of each sample was determined using the Bio-Rad DC protein assay kit (BIO-RAD, NSW, Australia). Array buffer 1 (blocking buffer: 2 mL) was added into each well of an 8-Well Multi-dish and array membranes were placed into each well. Membranes were incubated for 30 min at RT on a rocking platform shaker. After 30 min, the blocking buffer was removed and cell lysate samples (200 μg) were added and left shaking at 4 °C overnight. Membranes were washed 3 times with wash buffer I and wash buffer II, sequentially. Reconstituted detection antibody cocktail was added to each sample and incubated on a shaker for 1 h overnight at 4 °C. Following further washes, streptavidin-horse radish peroxidase was added and incubated on a shaker for 2 h at RT. Following further washes, chemi reagent mix was added and chemiluminescent images were captured using Syngene G-BOX (G: BOX-CHEMI-XL1.4, USA) and analysed using GeneTools Syngene software.

### Data analysis

Data is presented as mean ± standard error mean (SEM) and statistical analyses by Kruskal-Wallis nonparametric test followed by Dunn’s multiple comparisons test using GraphPad Prism 8.4.2 software. *P* < 0.05 was considered statistically significant.

## Results

### Assessing the apoptotic effect of Daunorubicin – Annexin V-FITC / PI assay

**Fig. 1:**
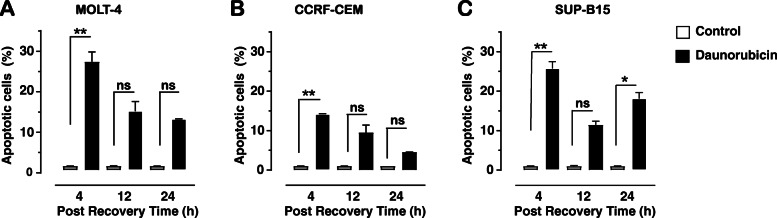
Percentage of apoptotic cells (Annexin-V positive) following daunorubicin treatment (10 μM) after recovery times of 4, 12 and 24 h for MOLT-4 (**a**), CCRF-CEM (**b**) and SUP-B15 (**c**) cells. Graph indicates mean of total of six replicates ± SEM, from three independent experiments. * (*p* < 0.05) and ** (*p* < 0.01) indicates significant difference between control and treatment and each time point using Kruskal-Wallis test followed by Dunn’s s multiple comparison test, while ns indicates no significant difference

Daunorubicin treatment causes induction of apoptosis, with each cell line showing different sensitivity and recovery patterns (Fig. 1). In MOLT-4 cells, apoptosis increased significantly after 4 h recovery (27.48% ± 2.46; *p* = 0.019, Fig. 1a) compared to the control. Following further incubation in the recovery medium for 12 h, levels of apoptosis declined compared to 4 h (14.88% ± 2.45) but remained at a comparable level after 24 h recovery (12.88% ± 0.10).

CCRF-CEM cells show a similar response to daunorubicin treatment to MOLT-4 cells. When CCRF-CEM cells were incubated for 4 h in recovery medium, there is a significant increase in apoptosis (14.15% ± 0.15; *p* = 0.005; Fig. 1b) compared to the control. The level of apoptosis gradually decreases from 14.15% (4 h recovery) to 4.51% ± 0.04 after 24 h incubation.

Treatment of SUP-B15 cells with daunorubicin results in a biphasic change in apoptotic levels. Initially a significant increase in apoptosis (25.75% ± 1.74; *p* = 0.002) occurs after 4 h recovery compared to control (Fig. 1c). After 12 h in recovery medium, there was a decrease relative to 4 h, but still higher than control levels (11.45% ± 1.61 *p* = 0.556). After 24 h in recovery medium, apoptotic levels increase again (18.11% ± 1.53; *p* < 0.041).

### General Caspase activation - Apostat assay

**Fig. 2:**
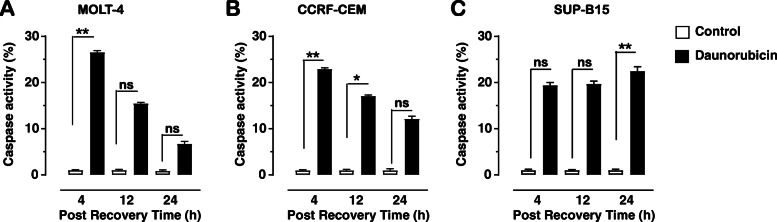
Percentage of cells containing active caspases (Apostat assay) following daunorubicin (10 μM) treatment and recovery times of 4, 12 and 24 h. MOLT-4 (**a**), CCRF-CEM (**b**) and SUP-B15 (**c**) cells. Graph indicates mean of total of six replicates ± SEM, from three independent experiments. * (*p* < 0.05) and ** (*p* < 0.01) indicates significant difference between control and treatment and each time point using Kruskal-Wallis test followed by Dunn’s s multiple comparison test, while ns indicates no significant difference

The apoptotic process is stimulated within 4 h after treatment, and to confirm if this is through caspase dependant mechanisms, assessment of caspase activation was conducted using Apostat. Both MOLT-4 and CCRF-CEM cells display initial activation of caspases after 4 h recovery, declining thereafter, but remaining elevated compared to the control. MOLT-4 cells exhibit a significant increase in caspase activity after 4 h recovery (26.54% ± 0.37; *p* = 0.002), compared to the control (Fig. 2a). After 12 h in recovery medium a decrease in caspase activity compared to the activity after 4 h occurs, but this is slightly elevated compared to control levels (15.48% ± 0.21; *p* = 0.051). The caspase activity continues to decline back to control levels after 24 h recovery (6.74% ± 0.48; *p* = 0.560).

Similarly, CCRF-CEM cells exposed to daunorubicin, showed a significant increase in caspase activity after 4 h recovery (22.97% ± 0.27; p = 0.002) compared to the control (Fig. 2b). Following 12 h in recovery medium, the caspase activity gradually declines compared to after 4 h recovery but is still significantly higher than the time relevant control (17.06% ± 0.23; *p* = 0.042) and is similar to control levels after 24 h (12.50% ± 0.56; *p* = 0.598).

SUP-B15 cells displayed a different pattern of caspase activation. After 4 h and 12 h recovery, there is a statistically insignificant increase in caspase activity (19.40% ± 0.62; *p* = 0.128 at both time points) compared to the control (Fig. 2c). The caspase activity increases further after 24 h (22.47% ± 0.94; *p* = 0.005).

### Changes in mitochondrial membrane potential - DiOC6 assay

**Fig. 3:**
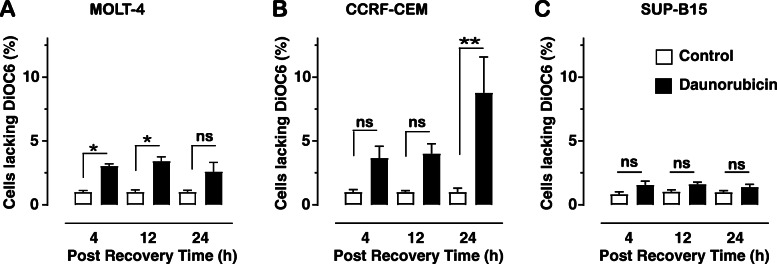
Percentage of cells lacking the DiOC6 dye following daunorubicin (10 μM) treatment and various recovery times, 4, 12 and 24 h. MOLT-4 (**a**), CCRF-CEM (**b**) and SUP-B15 (**c**) cells. The graph shows the percentage of cells lacking the DiOC6 dye indicative of decreases in mitochondrial Δψm. Graph indicates mean of total of six replicates ± SEM, from three independent experiments. * (*p* < 0.05), indicates significant difference between control and treatment at each time point using Kruskal-Wallis test followed by Dunn’s s multiple comparison test, while ns indicates no significant difference

Activation of caspases, impacting on apoptotic changes, can be initiated via two independent pathways, the intrinsic pathway involving the mitochondria and via the extrinsic death receptor pathways. To determine the involvement of mitochondria and thus the intrinsic pathway on caspase activation, changes in mitochondrial membrane potential (Δψm) were assessed using the DiOC6 assay. DiOC6 detects Δψm by accumulating in the mitochondrial matrix due to their large negative membrane potential, as the Δψm decreases less DiOC6 dye is retained. When MOLT-4 cells were treated with 10 μM daunorubicin (Fig. 3a) there was a significant decrease in the percentage of cells retaining the DiOC6 dye, indicating a decrease in Δψm after 4 h (3.05% ± 0.18 cells lacking DiOC6; *p* = 0.045) and 12 h in recovery medium (3.42% ± 0.33; *p* = 0.010). The decreased levels remaining after 24 h in recovery medium (2.60% ± 0.71; *p* = 0.413) were not significantly different to the control.

Treatment of CCRF-CEM cells with daunorubicin (Fig. 3b) resulted in a similar percentage of cells lacking the DiOC6 dye compared to the controls at 4 h and 12 h recovery (3.67% ± 0.90; *p* = 0.688 and 4.01% ± 0.76; *p* = 0.118) However, after 24 h recovery there was a significant increase in the percentage of cells lacking the DiOC6 dye, indicating a decrease in Δψm (7.53% ± 1.68; *p* = 0.010).

SUP-B15 leukemic cells displayed a different pattern of Δψm response to daunorubicin treatment (Fig. 3c). Following daunorubicin treatment of SUP-B15 cells, there is no significant increase in Δψm at any time point assessed, after 4 h recovery (1.90% ± 0.42), 12 h (2.10% ± 0.29), or 24 h recovery (1.81% ± 0.41).

### Apoptotic array

To decipher the apoptotic pathway, intrinsic or extrinsic, utilised by each of the cell lines CCRF-CEM, SUP-B15 and MOLT-4 following treatment with daunorubicin, the Abcam Human Apoptosis Antibody Array was utilised. This array allowed investigation into the effect the treatment had on multiple proteins involved in the apoptotic pathways. Changes were assessed after the initial 4 h treatment with daunorubicin only, cells were then allowed to recover in medium only for 12 h. Only select proteins involved in the intrinsic or extrinsic pathways were analysed and presented in Table 1, for full membrane analysis refer to Additional File 1: Figs. S1, S2 and Table S1.

**Table 1:** Changes in levels of apoptosis related protein in leukemic cell lines

	MOLT-4		CCRF-CEM		SUP-B15	
Apoptotic protein	DNR 4 h	Recovery Medium 12 h	DNR 4 h	Recovery Medium 12 h	DNR 4 h	Recovery Medium 12 h
Intrinsic Pathway						
p53	↑	–	↑	–	–	–
Bad	↑	–	↑	–	↓	–
Bax	↑	↑	↑	–	↓	↓
Bcl-2	↓	–	↓	↓	↑	↑
Bcl-w	–	–	↓	↓	↑	↑
cytC	↑	↑	↑	↑	↓	↓
SMAC	↑	↑	↑	–	–	–
Extrinsic Pathway						
Fas	↑	–	↑	–	↑	↑
FasL	↑	–	↓	↓	↑	–
sTNF-R1	↑	–	↑	↓	↓	↓
TRAIL-3	↑	–	↑	–	↑	↑
TNF-α	↑	–	–	↓	↑	–
CD40	↑	–	↑	↓	↑	↑
Casp-8	↑	↑	↑	↑	↑	↑
Casp-3	↑	↑	↑	↑	↑	↑

MOLT-4, CCRF-CEM, and SUP-B15 treated with 10 μM daunorubicin for 4 h and allowed 12 h recovery in drug-free medium

Following treatment and recovery, there were significant changes in apoptotic proteins, in both the intrinsic and extrinsic pathways, favouring apoptosis detected in MOLT-4 and CCRF-CEM cells. Increase in p53, and the pro-apoptotic Bad and Bax, and a decrease in the anti-apoptotic Bcl-2, stimulates mitochondrial changes along the intrinsic pathway. Changes in the mitochondrial membrane to release cytochrome c and second mitochondria-derived activator of caspases (SMAC), both of which levels increased, were also observed after 4 h. After 12 h in recovery medium the increased levels of Bax to continue to stimulate the mitochondrial membrane and subsequently both cytochrome c and SMAC levels remained elevated. Proteins involved in the extrinsic pathway also altered to favour apoptosis, with several death receptors, Fas and TNF-R1, and their ligands increasing in expression after the initial 4 h treatment. However, after 12 h in recovery medium, there was no change in receptor or ligand expression. The initiator caspase 8 and the executioner caspase 3 expression levels were increased at all times assessed. In addition, CCRF-CEM also displayed decreases in the anti-apoptotic proteins Bcl-2 and Bcl-w, further promoting apoptosis. Death receptor expression levels persisted after 12 h in recovery medium.

In contrast, changes in the expression levels of apoptotic proteins in SUP-B15 cells following daunorubicin treatment favoured the extrinsic pathway, rather than the intrinsic. The array shows a decrease in Bax protein, while an increase in the anti-apoptotic Bcl-2 and Bcl-x proteins, this would reduce the ability of Bax to initiate changes in the mitochondrial membrane. Without these changes, mitochondrial proteins, including cytochrome c, cannot be released which is shown to decrease in the array data. TNF-related apoptosis-inducing ligand (TRAIL), CD40, a member of the TNFR superfamily, and TNF-α increased after the 4 h treatment with daunorubicin, while Fas levels increased after the 12 h recovery. An increase in caspase 8 and caspase 3 occurred at all times assessed.

## Discussion

Leukaemia is a very heterogeneous disease, as such the response of different cancer types is going to vary greatly depending on genetic background and other functional changes within cells. To maximise and individualise treatment for any patient we must understand how these responses might impact on the cytotoxicity of treatments, including daunorubicin, and how cells may respond to treatments that contribute to chemotherapy resistance in the future. This study shows there are significant differences in how three leukaemia cell lines respond to daunorubicin treatment. The study focused on acute lymphoblastic leukaemia (ALL), MOLT-4 and CCFR-CEM (Acute T lymphoblastic leukaemia) and SUP-B15 (Acute B lymphoblastic leukaemia) cell lines as daunorubicin is widely used in the treatment of this leukaemia [27] and the process of lymphoid tumorigenesis often involves alterations to the ataxia-telangiectasia mutated (ATM) gene resulting in ATM deficient cells which are more sensitive to oxidative stress and are likely to undergo altered DNA repair and apoptotic pathways [28]. There are differences in the level of response in each cell line, and how long and how effective that response ultimately was. In MOLT-4 and CCRF-CEM, daunorubicin induced apoptosis via both the intrinsic (mitochondrial-mediated) and extrinsic (death-receptor mediated) pathways. In MOLT-4 and CCRF-CEM cells, daunorubicin induced apoptosis via both the intrinsic (mitochondrial-mediated) through loss of Δψm, activation of caspases and down regulation of Bcl-2, and extrinsic (death-receptor mediated) pathways occurs via triggering death receptors such as Fas, CD40, TRAIL-3 and TNF-α. However, in SUP-B15, apoptosis was induced in two waves, corresponding to caspase activation, without changes in Δψm; and an increase in death receptor proteins suggesting only extrinsic apoptosis was induced. These differences have not previously been reported as this study compares leukaemia’s stemming from different cell linages: MOLT-4 and CCRF-CEM are acute T-lymphoblastic leukaemia; while SUP-B15 is an acute B-lymphoblastic leukaemia cells. We have previously shown differences in response to daunorubicin through activation of p53, a key regulator of cellular responses to DNA damage caused by the treatment, where p53 was phosphorylated at serine 15 only in MOLT-4 and CCRF-CEM, but not in SUP-B15 cells [25, 29]. The lack of functional p53 in SUP-B15 cells may have contributed to the altered mechanism and more prolonged apoptotic response.

Daunorubicin induces apoptosis in ALL cell lines but with distinct variations. Apoptosis was induced in MOLT-4 and CCRF-CEM cell lines as early as 4 h post treatment. We have previously reported daunorubicin induced toxic reactive oxygen species and DNA double strand breaks in MOLT-4 and CCRF-CEM cell lines after 4 h [25]. In addition, a study conducted on MOLT-4 cells and human acute myeloblastic leukaemia (ML-1) cells demonstrated that daunorubicin significantly enhanced DNA fragmentation indicating apoptosis [2]. A further study in acute myelogenous leukemia, specifically acute promyelocytic leukemia (HL60) and chronic myelogenous leukemia (K562) leukemic cells, demonstrated that daunorubicin displayed a time and concentration dependant cytotoxic effect. As early as 2 h treatment, but the higher concentration (7.0 μM) lead to rapid degradation of DNA, indicating apoptosis after a shorter time [5]. A different pattern was seen in the levels of apoptosis in SUP-B15 cells following daunorubicin treatment. Although apoptosis levels initially increase comparably, they remain elevated over the times examined. We previously reported a delay in DNA double strand break repair and lower sensitivity to daunorubicin in the B lymphocyte derived SUP-B15 cells, potentially due to dysfunctional p53 causing variation in the DNA repair pathways [25] which would alter the signalling mechanism and potentially prolong apoptosis.

Results from the apoptotic array only indicated changes in the levels of each protein in response to daunorubicin. However, it is not just an increase, or decrease in these proteins that can trigger apoptosis, rather activation or interaction of certain proteins that determines the cells fate. Using the apoptosis array provides an initial overview of the levels of proteins, since anthracyclines have been shown to transcriptionally regulate apoptotic proteins, that can potentially decrease their effectiveness over time and contribute to chemoresistance [30]. Further experiments would need to be done to confirm which areas of the cells these proteins are found and what proteins they are interacting with, to enable a better understanding of the apoptotic response. In the current study, changes in apoptotic proteins in SUP-B15 cells favours the extrinsic pathway for apoptosis following treatment with daunorubicin, while inhibiting the intrinsic pathways. Following treatment, the increase in the expression of Bcl-2 and Bcl-w in SUP-B15 cells is likely to inhibit the action of Bax altering the mitochondrial membrane permeability and thus decreasing the involvement of the mitochondrial pathway. Also, mutations in p53, a crucial regulator of the intrinsic pathway was shown to be dysfunctional in SUP-B15 [25], and thus unable to increase Bax levels, and inhibit the expression Bcl-2 and Bcl-w, both of which increased in this study. Transcriptional upregulation of Bcl-2 in response to daunorubicin treatment has previously been demonstrated in leukaemia cell lines and plays a key role in increased resistance to chemotherapies [31]. Changing the levels of Bcl-2 and Bax in cells exposed to daunorubicin will alter the balance between the anti- and pro-apoptotic proteins [32]. These changes would inhibit apoptosis via the intrinsic pathways.

Daunorubicin is known to lead to the activation of effector, or downstream caspases (mainly caspase 3), responsible for the cleavage of crucial substrates in the degradation phase of cell death [5, 33]. Total activated caspases were investigated with the Apostat assay and it was found that all three ALL cell lines showed significant increase in activated caspases after treatment. Although the ApoStat assay allowed us to gauge the overall levels of caspase activity, it did not distinguish between the specific subgroups of caspases involved. Even though the array did confirm an increase in caspase 8, involved in the extrinsic pathway, and caspase 3, the executioner caspase, in all cell lines, the levels of caspase 9, involved in the intrinsic pathway was not included in the array. Similar to these results, doxorubicin a closely related anthracycline, has previously been shown to stimulate an increase in activated caspases 8 and 9 [34]. The activation of caspase 8, as opposed to caspase 9, has a huge impact on the cellular injury sequelae that ensues [35].

Mitochondria play an essential role in the induction of apoptosis via the intrinsic pathway. An important event in the mitochondrial apoptotic pathway is mitochondrial outer membrane permeabilization, which is primarily mediated and controlled by the Bcl-2 family members [36, 37]. The present study determined the effect of daunorubicin on several Bcl-2 family members, both pro-apoptotic and anti-apoptotic, and identified a change in MOLT-4 and CCRF-CEM cells to favour mitochondrial membrane changes that promote apoptosis. Consequently, a change in the mitochondrial membrane potential was also observed in these cells. There was a slight delay in mitochondrial changes in CCRF-CEM compared to MOLT-4, possibly due to a decrease in the levels of Bcl-w observed in CCRF-CEM, but not MOLT-4 cells. Decreased expression of Bcl-w may be a consequence of abnormal activation of the p53 signalling cascade due to missense mutations [38], but ultimately results in mitochondrial mediated apoptosis being initiated. Therefore, daunorubicin may trigger cell death via a p53-dependent and p53-independent pathway, supporting evaluation of the p53 status of a given patient’s leukemia cells in selection of chemotherapeutic agents.

Altered membrane potential facilitates the release of mitochondrial proteins including cytochrome c that induces apoptosis in both MOLT-4 and CCRF-CEM, but these changes were not observed in SUP-B15 cell line. This finding supports the view that alteration of mitochondria functions plays a major role in the apoptosis, in particular, in cell death induced by daunorubicin but reflects the different sensitivity of ALL types dependant on the expression profile of these proteins. The SUP-B15 cells did not display any significant changes to proteins involved in the intrinsic pathway, however they still displayed comparable levels of apoptosis and caspase activation. Results from the array indicate increased expression of death receptors and their natural ligands, including CD40, Fas, TRAIL and sTNF, following daunorubicin treatment. Binding to their corresponding ligands results in receptor aggregation and hence recruitments of intracellular proteins, for example FADD and pro-caspase 8 to aggregated receptor recruiting DISC [39–41]. Recruitment of DISC proteins such as FADD results in increase of caspase-8 which has been shown to activate caspase-3 [42, 43]. Increasing the expression levels of caspase-3 activity led to apoptosis. Results from the array indicate signalling within SUP-B15 potentially involved the extrinsic pathway, which would ultimately lead to the activation of caspase 8 and caspase 3, and subsequent apoptotic degradation after 4 h recovery. However, the second increase of apoptosis after 24 h recovery could involve caspase 8 cleavage of Bid into truncated Bid which could then activate the intrinsic pathway leading to apoptosis [44] but this would need to be further explored. Results from the array indicate a decrease in the levels of Bid, but do not indicate the level of activation.

## Conclusion

This study has identified varying sensitivity and mechanisms involved in cellular responses to daunorubicin in different leukaemia cell lines. MOLT and CCRF-CEM cells, the T-lymphoblastic leukaemia, responded by initiating both the intrinsic and extrinsic pathways resulting in significant apoptosis, mitochondria involvement and caspase activation. The level of apoptosis increased initially and subsided once damaged cells had been removed. In contrast, SUP-B15, the B-lymphoblastic leukaemia which lacks p53 activity, apoptosis stimulation was more prolonged, with both Annexin V expression and caspase activity more significant after 24 h in recovery medium, excluding any changes in the mitochondrial membrane potential. The results from this study have added to our current knowledge of how leukemic cell lines respond differently to daunorubicin, and hint as to why clinical effective doses vary between leukaemia types. Further work needs to be done is required to confirm the involvement of different apoptotic pathways apoptotic as potential targets to maximising cytotoxic treatments, and translate the results using an in vivo models.

## Supplementary Information

**Additional file 1: Fig. S1.** The array membrane template. **Fig. S2.** Example array panels demonstrating the levels of human apoptotic proteins in MOLT-4 lysates **a)** without treatment (control medium) **b)** DNR treated **c)** 12 h recovery medium. **Table S1.** Relative changes in levels of apoptosis related protein when treated with DNR for 4 h and 12 h in drug free medium (recovery medium) compared to medium only control.

## Data Availability

All data generated or analysed during this study are included in this article and its Additional file 1.

## Abbreviations

ALL: acute lymphoblastic leukaemia
ATM: ataxia-telangiectasia mutated
Δψm: mitochondrial membrane potential
ROS: reactive oxygen species
MOMP: mitochondrial outer membrane permeabilisation
BH: Bcl-2 homology domains
TNFR: TNF receptors
TRAIL-R1: Tumor necrosis factor-Related Apoptosis-Inducing Ligand - Receptor 1
DR3: Death Receptor 3
FADD: Fas Associated Death Domain
DISC: death-inducing signalling complex
BID: BH3-interacting domain death agonist
SMAC: second mitochondria-derived activator of caspases
TRAIL: Tumour necrosis factor-related apoptosis-inducing ligand
TNF: tumour necrosis factor
