# Supplementary material for: Intrinsic and extrinsic apoptosis responses in leukaemia cells following daunorubicin treatment
Source: BMC Cancer. 2021 Apr 21;21:438. doi: 10.1186/s12885-021-08167-y (PMC8059319; doi:10.1186/s12885-021-08167-y)
Supplement: Supplementary file 1 — Additional file 1: Fig. S1. The array membrane template. Fig. S2. Example array panels demonstrating the levels of human apoptotic proteins in MOLT-4 lysates a) without treatment (control medium) b) DNR treated c) 12 h recovery medium. Table S1. Relative changes in levels of apoptosis related protein when treated with DNR for 4 h and 12 h in drug free medium (recovery medium) compared to medium only control. [file 12885_2021_8167_MOESM1_ESM.pdf]

Additional File for

Intrinsic and extrinsic apoptosis responses in leukaemia cells following daunorubicin treatment

Hussain Mubarak Al-Aamri [1,2], Helen R Irving[1], Christopher Bradley[1] and Terri Meehan-Andrews[1]

[1] Department of Pharmacy and Biomedical Science, La Trobe Institute for Molecular Science (LIMS), La Trobe University, P.O. Box 199, Bendigo, VIC 3552, Australia

[2]Oman College of Health Sciences, PO Box 293, Postal code 620, Ruwi, Sultanate of Oman

The following pages include:

- Table S1
- Figures S1 and S2

	A	B	C	D	E	F	G	H	I	J	K	L	M	N
1	Pos	Pos	Neg	Neg	BLANK	BLANK	Bad	Bax	Bcl-2	Bcl-w	BID	BIM	Caspase 3	Caspase 8
2	Pos	Pos	Neg	Neg	BLANK	BLANK	Bad	Bax	Bcl-2	Bcl-w	BID	BIM	Caspase 3	Caspase 8
3	CD40	CD40L	cIAP-2	cytoC	DR6	Fas	FasL	BLANK	HSP27	HSP60	HSP70	HTRA	IGF-I	IGF-II
4	CD40	CD40L	cIAP-2	cytoC	DR6	Fas	FasL	BLANK	HSP27	HSP60	HSP70	HTRA	IGF-I	IGF-II
5	IGFBP-1	IGFBP-2	IGFBP-3	IGFBP-4	IGFBP-5	IGFBP-6	IGF-1sR	Livin	p21	p27	p53	SMAC	Survivin	sTNF-R1
6	IGFBP-1	IGFBP-2	IGFBP-3	IGFBP-4	IGFBP-5	IGFBP-6	IGF-1sR	Livin	p21	p27	p53	SMAC	Survivin	sTNF-R1
7	sTNF-R2	TNF-α	TNF-β	TRAIL R-1	TRAIL R-2	TRAIL R-3	TRAIL R-4	XIAP	BLANK	BLANK	Neg	Neg	Neg	Pos
8	sTNF-R2	TNF-α	TNF-β	TRAIL R-1	TRAIL R-2	TRAIL R-3	TRAIL R-4	XIAP	BLANK	BLANK	Neg	Neg	Neg	Pos

Figure S1. The array membrane template

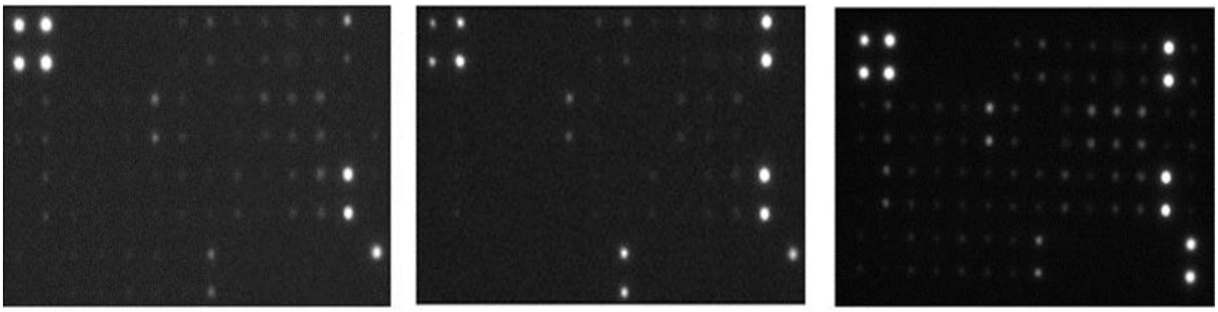

a) **b)** **c)**

Figure S2. Example array panels demonstrating the levels of human apoptotic proteins in MOLT-4 lysates **a)** without treatment (control media) **b)** DNR treated **c)** 12 hour recovery media

Table S1. Relative changes in levels of apoptosis related protein when treated with DNR for 4 hours and 12 hour in drug free media (recovery media) compared to media only control.

	MOLT-4		CCRF-CEM		SUP-B15	
	DNR 4hrs	Recovery Media 12hrs	DNR 4hrs	Recovery Media 12hrs	DNR 4hrs	Recovery Media 12hrs
**Bad**	↑	–	↓	–	↑	–
**Bax**	↑	↑	↓	↓	↑	–
**Bcl-2**	↓	–	↑	↑	↓	↓
**Bcl-w**	–	–	↑	↑	↓	↓
**Bid**	–	–	–	–	↓	↓
**Bim**	↓	–	–	–	↓	↓
**Casp-3**	↑	↑	↑	↑	↑	↑
**Casp-8**	↑	↑	↑	↑	↑	↑
**CD40**	↑	–	↑	↑	↑	↓
**CD40L**	–	–	↑	↑	–	↓
**cIAP-2**	↓	–	–	–	–	↓
**cytC**	↑	↑	↓	↓	↑	↑
**DR6**	↓	–	↓	–	↓	↓
**Fas**	↑	–	↑	↑	↑	–
**FasL**	↑	–	↑	–	↓	↓
**HSP27**	↓	–	–	–	↓	–
**HSP60**	↑	–	–	–	↑	–
**HSP70**	↓	↓	–	–	↓	↓
**HTRA2**	–	↓	↓	–	–	↓
**IGF-I**	↓	–	↑	↑	↓	↓
**IGF-II**	↓	–	↑	↑	↓	↓
**IGFBP-1**	–	–	–	↑	↑	↓
**IGFBP- 2**	↑	↑	↑	↑	↑	↑
**IGFBP- 3**	–	–	↑	–	↑	↓
**IGFBP- 4**	↓	↑	–	–	↑	↑
**IGFBP- 5**	↑	↑	–	–	↑	–
**IGFBP- 6**	↓	–	↑	–	–	↓
**IGF-1sR**	↓	↓	↑	–	↓	↓
**Livin**	↓	–	↑	–	–	↓
**P21**	↑	↓	↓	↓	↑	↓
**P27**	↑	↓	↓	–	↑	↑
**P53**	↑	↓	↓	↓	**-**	**-**
**SMAC**	↑	↑	–	–	↑	–
**Survivin**	–	↓	↑	↑	–	–
**sTNF-R1**	↑	–	↓	↓	↑	↓
**sTNF-R2**	–	–	↑	–	↑	↓
**TNF- α**	–	–	↑	–	–	↓
**TNF- β**	↓	↓	↑	–	↓	↓
**TRAILR-1**	–	–	↑	–	–	↓
**TRAILR-2**	↓	↓	↑	–	–	–
**TRAIL-3**	↑	–	↑	↑	↑	–
**TRAIL-4**	–	–	↑	↑	–	↓
**XIAP**	↓	–	↑	↑	↑	↓
